# Association Between Body Mass Index and Depression/Anxiety in an East Asian Population: A Mendelian Randomization Study

**DOI:** 10.1002/kjm2.70161

**Published:** 2025-12-24

**Authors:** Perl Han Lee, Szu‐Chia Chen, Chien‐Hung Lee, Jiun‐Hung Geng, Chih‐Hung Ko

**Affiliations:** ^1^ Graduate Institute of Medicine, College of Medicine Kaohsiung Medical University Kaohsiung Taiwan; ^2^ Department of Psychiatry Kaohsiung Medical University Hospital Kaohsiung Taiwan; ^3^ Faculty of Medicine, College of Medicine Kaohsiung Medical University Kaohsiung Taiwan; ^4^ Department of Internal Medicine, Kaohsiung Municipal Siaogang Hospital Kaohsiung Medical University Kaohsiung Taiwan; ^5^ Division of Nephrology, Department of Internal Medicine, Kaohsiung Medical University Hospital Kaohsiung Medical University Kaohsiung Taiwan; ^6^ Department of Public Health, Environmental Medicine Research Center, College of Health Sciences Kaohsiung Medical University Kaohsiung Taiwan; ^7^ Graduate Institute of Clinical Medicine, College of Medicine Kaohsiung Medical University Kaohsiung Taiwan; ^8^ Research Center for Environmental Medicine Kaohsiung Medical University Kaohsiung Taiwan; ^9^ Department of Urology Kaohsiung Municipal Siaogang Hospital Kaohsiung Taiwan; ^10^ Department of Urology, Kaohsiung Medical University Hospital Kaohsiung Medical University Kaohsiung Taiwan; ^11^ Department of Urology, School of Medicine, College of Medicine Kaohsiung Medical University Kaohsiung Taiwan; ^12^ Department of Psychiatry Kaohsiung Municipal Siaogang Hospital Kaohsiung Taiwan

**Keywords:** anxiety, body mass index, depression, Mendelian randomization

## Abstract

Obesity and psychiatric disorders are the leading causes of global morbidity. Epidemiological studies suggest a bidirectional link between higher body mass index (BMI) and mental health outcomes, but the direction of causality remains uncertain due to confounding and reverse causation. We performed a Mendelian randomization (MR) analysis using genetic instruments for BMI (13 and 17 single‐nucleotide polymorphisms [SNPs] identified in the Taiwan Biobank) from 107,191 Taiwanese adults (4855 “cases” with depression or anxiety and 102,336 “controls”) to examine the association between BMI and depression and anxiety symptoms. The primary MR results revealed an inverse causal association between the two. An inverse‐variance weighted analysis using 17 SNPs indicated that each one‐unit increase in genetically predicted BMI was associated with a lower risk of depression and anxiety symptoms. Analyses restricted to 13 strong BMI‐associated SNPs revealed a similar inverse direction of effect. The results suggest that higher BMI may be associated with a reduced risk of psychiatric disorders in this East Asian population, highlighting potential differences by population or underlying mechanism. These findings demonstrate how MR can clarify complex exposure–outcome relationships and highlight the need to explore the biological and psychosocial pathways connecting adiposity and mental health.

## 1 Introduction

The global rise in obesity has drawn growing attention to its impact not only on physical health but also on mental well‐being. Body mass index (BMI) has been associated with a range of psychiatric conditions, including depression and anxiety disorders [1, 2, 3]. A meta‐analysis of 25 studies revealed that overweight and obesity were significantly associated with a higher frequency of anxiety [4]. Another meta‐analysis of eight longitudinal studies reported that obesity was associated with an increased risk of depression [5]. This association seems to be bidirectional: individuals with obesity have a 55% increased risk of depression, whereas individuals with depression have a 58% higher likelihood of becoming obese [6]. Despite these close connections between obesity and psychiatric disorders, many studies are limited by confounding factors and the possibility of reverse causality, particularly since psychiatric disorders can influence lifestyle behaviors, such as diet and physical activity, which in turn affect BMI [7, 8].

Several biological and psychosocial pathways could contribute to the observed relationship between BMI and depression/anxiety symptoms. Excess adiposity induces chronic systemic inflammation, which may adversely affect brain function and mood [6]. Metabolic disturbances related to obesity (e.g., insulin resistance or dysregulated hypothalamic–pituitary–adrenal axis) have also been implicated in depression and anxiety [9]. Furthermore, individuals with obesity often experience stigma and body image dissatisfaction, which can exacerbate depression and anxiety [10]. Conversely, certain psychiatric conditions can promote weight gain due to behavioral changes or medication side effects. Disentangling these effects is challenging because confounding factors (e.g., socioeconomic status, diet, or comorbid illnesses) and reverse causation (e.g., depression leading to reduced physical activity and subsequent weight gain) can bias estimates of the obesity–mental health relationship.

Mendelian randomization (MR) is a powerful tool for causal inference. By using genetic variants as instrumental variables and leveraging the random allocation of alleles at conception, MR mimics a natural randomized controlled trial and can help reduce bias from confounding and reverse causation [11, 12]. Previous MR studies in Western populations have produced mixed findings regarding the effect of BMI on mental health outcomes [13, 14], and few studies have examined this relationship in East Asian populations.

In this study, we used MR to examine the association between BMI and depression/anxiety symptoms in an Asian population. Using data from the Taiwan Biobank, we investigated whether genetically predicted BMI has a causal effect on depression and anxiety symptoms. Our findings are expected to not only clarify the causality in this relationship but also inform future strategies for integrated physical and mental health care.

## 2 Materials and Methods

### 2.1 Taiwan Biobank and Study Design

The Taiwan Biobank is a comprehensive human biological database comprising a cohort of more than 100,000 Taiwanese individuals aged 30–70 years. It collects data on biological markers, environmental factors, and lifestyle habits to support research aimed at identifying strategies to address the onset, progression, and treatment of diseases and to enhance public health [15, 16, 17]. After quality control, 107,191 unrelated participants of Han Chinese ancestry with available genetic and phenotypic data were included in the analysis. The study was approved by the Institutional Review Board of Taiwan Biobank.

### 2.2 Phenotype Definitions

Depression and anxiety symptoms were considered as a binary variable. A participant who met any of the following criteria was coded as a “case”:

1. A self‐reported diagnosis of depression;
2. A Generalized Anxiety Disorder‐2 (GAD‐2) scale score > 3; or
3. A Patient Health Questionnaire‐2 (PHQ‐2) scale score > 3.

An individual was considered as having depression if they responded to a simple “yes”/“no” question: “Have you ever been diagnosed with depression”? Participants answering “yes” were further asked, “When were you diagnosed with depression?”

Both GAD‐2 scale items are rated on a 4‐point Likert scale ranging from 0 (*not at all*) to 3 (*almost every day*), focusing on the emotional and cognitive expressions of DSM‐IV anxiety [18]. It has shown good internal consistency (*ω* = 0.80) [19]. The PHQ‐2 scale items are also rated on a Likert scale; the scale focuses on the core symptoms of depression (anhedonia and depressed mood) [20]. The PHQ‐2 was validated and showed adequate levels of internal consistency (α = 0.80) [21].

Participants who did not meet any of these criteria were classified as “controls.”

### 2.3 Genotyping and Quality Control

Genotyping was conducted using a customized Axiom‐Taiwan Biobank Array Plate. Standard quality control procedures were applied, including the exclusion of single‐nucleotide polymorphisms (SNPs) with call rates < 98%, minor allele frequency (MAF) ≤ 5%, or Hardy–Weinberg equilibrium *p* < 1 × 10^−6^. Participants with cryptic relatedness, sex mismatch, or genotype call rates < 95% were also excluded.

### 2.4 Genetic Instrument Selection for BMI

A global large‐scale genome‐wide association study (GWAS) of approximately 718,000 individuals has identified hundreds of genetic loci associated with BMI [22]. GWAS was also conducted within the Taiwan Biobank to identify variants associated with BMI. We cross‐referenced the BMI‐associated SNPs reported by that study with variants available in the Taiwan Biobank, limiting them to those with a MAF ≥ 5%. *F* statistics were calculated for each SNP to assess instrument strength, and SNPs with *F* > 5 were selected to minimize weak instrument bias. A subset of 13 SNPs with *F* > 5 was retained as robust instruments for secondary MR analysis.

### 2.5 MR Analysis

A one‐sample MR design was employed using individual‐level data from the same cohort for both exposure (BMI) and outcome (depression and anxiety symptoms). We adjusted the GWAS for BMI for potential population stratification by including age, sex, and the top 10 principal components as covariates. We conducted MR analyses using multiple MR methods, primarily inverse‐variance weighted (IVW), with sensitivity analyses using simple median, weighted median, MR‐Egger, and outlier‐robust approaches (penalized weighted median and penalized IVW). These methods were selected a priori to test the consistency of the causal estimate under different assumptions. All MR estimates were reported with standard errors (SEs), 95% confidence intervals, and *p* values. MR‐Egger intercepts were tested to detect horizontal pleiotropy.

### 2.6 Statistical Analyses

Descriptive statistics comparing cases and controls are presented as means with standard deviations for continuous variables and proportions for categorical variables. Between‐group comparisons were made using *t*‐tests or chi‐square tests, as appropriate. MR analyses were conducted using the R package *MendelianRandomization* version 0.10.0, and custom scripts were used to compute *F* statistics and conduct sensitivity analyses.

## 3 Results

### 3.1 Participant Characteristics

We included 107,191 participants (4855 cases and 102,336 controls) from the Taiwan Biobank (Table 1). Compared with the controls, the case group included more women, had higher rates of tobacco use, and reported greater exposure to environmental tobacco smoke. Metabolic syndrome was more prevalent in cases (25.6% vs. 22.4%, *p* < 0.001; Table 1). No significant differences were observed in mean age. The mean BMI was similar between cases and controls (24.2 vs. 24.2, *p* = 0.327; Table 1), suggesting no significant association in the unadjusted analyses. However, observational associations can be confounded or biased by reverse causation. Using data from the Taiwan Biobank, a recent study constructed polygenic risk scores for both BMI and major depressive disorder to evaluate their effects on obesity‐related traits [23]. The study provided evidence of a shared genetic foundation between obesity and depression. Here, we performed MR to test for a causal effect of BMI on depression and anxiety symptoms.

**TABLE 1:** Clinicodemographic characteristics of the study participants.

Characteristics	Total (*N* = 107,191)	Control (*N* = 102,336)	Case (*N* = 4855)	*p*
Sex, *n* (%)				< 0.001***
Male	38,571 (36.0%)	37,340 (36.5%)	1231 (25.4%)	
Female	68,620 (64.0%)	64,996 (63.5%)	3624 (74.6%)	
Age, mean (SD)	49.9 (10.9)	49.9 (10.9)	50.0 (10.7)	0.4508
Age group, *n* (%)				0.0011**
30–39	24,082 (22.5%)	23,066 (22.5%)	1016 (20.9%)	
40–49	25,639 (23.9%)	24,460 (23.9%)	1179 (24.3%)	
50–59	32,382 (30.2%)	30,800 (30.1%)	1582 (32.6%)	
≥ 60	25,088 (23.4%)	24,010 (23.5%)	1078 (22.2%)	
Tobacco use^a^, *n* (%)				< 0.001***
No	78,004 (72.8%)	74,664 (73.0%)	3340 (68.8%)	
Yes	29,187 (27.2%)	27,672 (27.0%)	1515 (31.2%)	
Exposure to ETS, *n* (%)				< 0.001***
No	95,885 (89.5%)	91,661 (89.6%)	4224 (87.0%)	
Yes	11,306 (10.5%)	10,675 (10.4%)	631 (13.0%)	
BMI, mean (SD)	24.2 (3.77)	24.2 (3.77)	24.2 (4.07)	0.3271
Metabolic syndrome, *n* (%)				< 0.001***
No	83,028 (77.5%)	79,414 (77.6%)	3614 (74.4%)	
Yes	24,163 (22.5%)	22,922 (22.4%)	1241 (25.6%)	

Abbreviations: BMI, body mass index; ETS, environmental tobacco smoke.

^a^ Defined as at least 6 months.

* *p* < 0.05.

** *p* < 0.01.

*** *p* < 0.001.

### 3.2 Instrument Selection

We selected 36 BMI‐associated SNPs from the Taiwan Biobank dataset that had an MAF of ≥ 5% (Table 2). Of them, 17 variants were significantly associated with BMI (*p* < 0.05) and were selected as candidate instruments (Table 2).

**TABLE 2:** GWAS catalog for variants selection associated with body mass index (36 variants).

ID	*β*	SE	*p*	REF	ALT	A1	Omitted
rs1801265	−0.0393553	0.0315236	0.211872	A	G	G	A
rs62623713	−0.00516366	0.184807	0.977709	A	G	G	A
rs2297792	0.102472	0.0254255	5.57509 × 10^−5^ ***	T	C	C	T
rs591120	0.0818076	0.0178911	4.82447 × 10^−6^ ***	G	C	C	G
rs4851287	0.0382519	0.0378785	0.312566	G	A	A	G
rs2230590	0.0820724	0.0235234	0.000485096***	T	C	C	T
rs56384862	0.291985	0.231554	0.207318	A	G	G	A
rs1052618	−0.0504323	0.0240737	0.0361814*	G	A	A	G
rs9438	0.03299	0.0163816	0.0440287*	G	C	C	G
rs459552	0.0251258	0.0270626	0.353185	A	T	T	A
rs11755393	0.0430231	0.0163265	0.00841049**	A	G	G	A
rs1539172	0.00746285	0.0171038	0.662601	A	G	G	A
rs2280843	−0.00114376	0.0182766	0.950101	G	A	A	G
rs10829163	−0.0385399	0.0164266	0.0189681*	T	C	C	T
rs3088142	−0.0497489	0.0280689	0.0763332	T	C	C	T
rs284860	−0.0438767	0.0167741	0.00890461**	T	C	C	T
rs11042023	0.0385567	0.0166024	0.0202156*	T	C	C	T
rs11555762	0.0452776	0.0185979	0.0149116*	C	T	T	C
rs1064608	0.0700331	0.0179628	0.000096739***	G	C	C	G
rs12828016	−0.0399526	0.0181992	0.028145*	G	T	T	G
rs3184504	−0.0396742	0.332219	0.904942	C	T	T	C
rs1169081	0.0203762	0.0162857	0.210876	T	G	G	T
rs1131877	0.0529513	0.0166897	0.00151079**	T	C	C	T
rs11071896	−0.045091	0.0216912	0.0376408*	A	G	G	A
rs2277598	0.0312289	0.0190052	0.100349	T	C	C	T
rs4077410	0.101141	0.0163949	6.89319 × 10^−10^ ***	A	G	G	A
rs3213758	0.00936643	0.0174651	0.591756	C	T	T	C
rs1071648	−0.00926889	0.0215475	0.66708	T	C	C	T
rs2306590	−0.0694435	0.0177071	8.79495 × 10^−5^ ***	G	A	A	G
rs9891146	−0.0954524	0.0183206	1.89091 × 10^−7^ ***	T	C	C	T
rs3760128	0.00800899	0.0203709	0.694203	A	G	G	A
rs2396359	−0.0182009	0.0165373	0.271075	T	C	C	T
rs2075803	0.00549412	0.0164372	0.738192	G	A	A	G
rs2228273	0.015805	0.0253	0.532168	G	A	A	G
rs2076559	−0.0118632	0.0168664	0.481831	A	G	G	A
rs5758651	−0.0266672	0.0170116	0.11698	T	C	C	T

* *p* < 0.05.

** *p* < 0.01.

*** *p* < 0.001.

**FIGURE 1:**
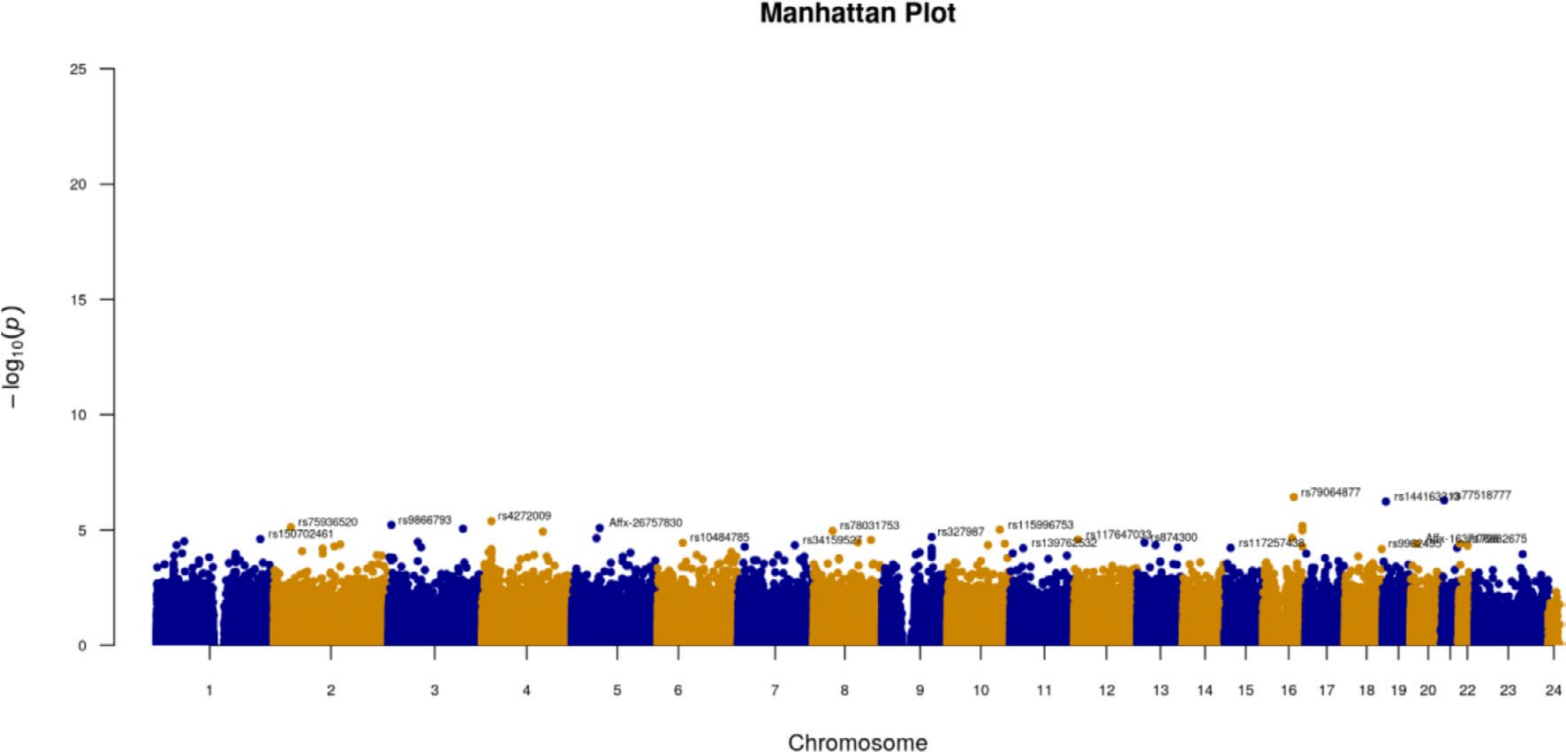
Manhattan plot of genome‐wide association study on depression/anxiety.

**FIGURE 2:**
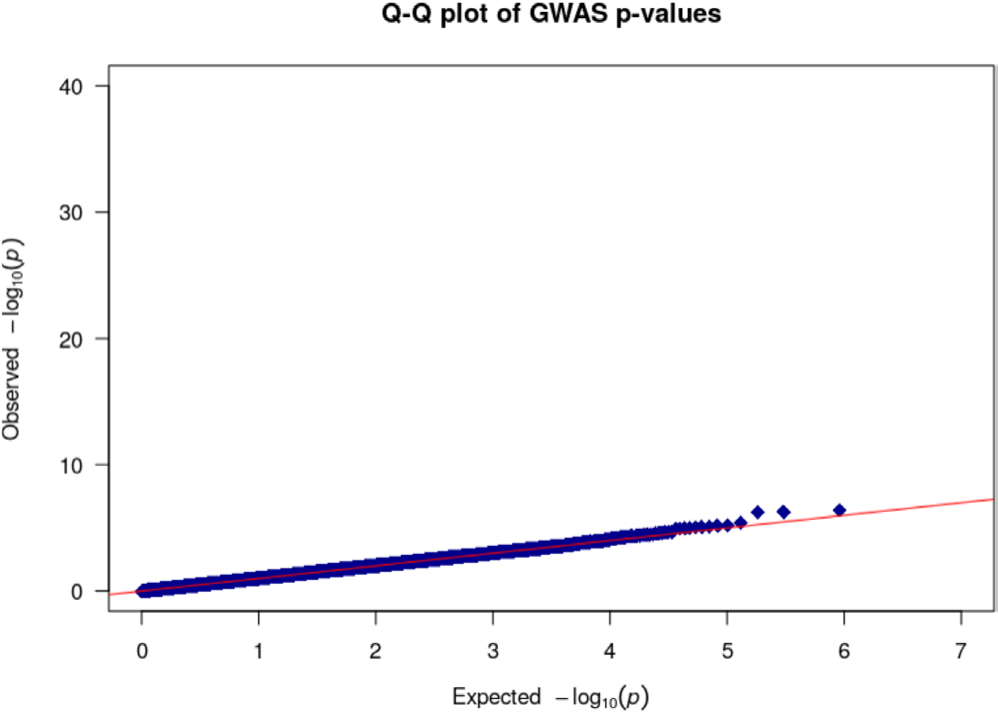
Quantile–quantile (QQ) plot of the data shown in the Manhattan plot.

The Manhattan plot and quantile–quantile (QQ) plot are presented in Figures 1 and 2, respectively. The QQ plot demonstrated minimal deviation from the expected null distribution, indicating a good overall model fit. The genomic inflation factor (*λ*) was 1.016, suggesting negligible population stratification or systemic inflation of the test statistics.

*F* statistics were calculated for each variant (Table 4). Many MR studies use *F* > 10 to minimize weak instrument bias. However, due to the smaller effect sizes in an Asian population, using *F* > 10 would leave very few instruments (eight SNPs), which would considerably lower the statistical power; consequently, methods such as MR‐Egger became very unstable with wide CIs and high SEs. Considering that most MR studies prefer using at least 10–15 well‐clumped SNPs, we used *F* > 5 as a compromise. Therefore, 13 SNPs with *F* > 5 were retained as strong instruments for the refined MR analysis.

### 3.3 Primary MR Analysis

Conducted on all 17 significant SNPs, an IVW MR analysis revealed a significant inverse association between BMI and depression/anxiety symptoms (*β* = −0.8746, SE = 0.3580, 95% CI: −1.5762 to −0.1729, *p* = 0.0146) (Table 3). Penalized IVW and robust IVW, which reduce the influence of outlier SNPs and improve robustness, produced significant results with narrower confidence intervals and smaller *p* values (e.g., penalized IVW: *β* = −1.1905, *p* = 0.00089). Median‐based estimators (simple, weighted, and penalized weighted medians) also yielded negative estimates (*β* = −1.19), consistent with the IVW direction. The MR‐Egger intercepts indicated no evidence of horizontal pleiotropy (*p* = 0.0897–0.4112).

**TABLE 3:** Mendelian randomization using selected 17 variants with *p* < 0.05 in Taiwan Biobank population.

Method	Estimate	SE	95% CI		*p*
Simple median	−1.18906	0.52264	−2.21341	−0.16471	0.02290*
Weighted median	−1.12799	0.49823	−2.10451	−0.15147	0.02358*
Penalized weighted median	−1.18528	0.49696	−2.15930	−0.21125	0.01708*
IVW	−0.87455	0.35800	−1.57621	−0.17289	0.01457*
Penalized IVW	−1.19045	0.35825	−1.89260	−0.48829	0.00089***
Robust IVW	−1.33395	0.47163	−2.25833	−0.40958	0.00468**
Penalized robust IVW	−1.26803	0.44770	−2.14551	−0.39055	0.00462**
MR‐Egger	1.65825	1.53052	−1.34152	4.65803	0.27861
Intercept	−0.04309	0.02539	−0.09286	0.00667	0.08966
Penalized MR‐Egger	1.65825	1.53052	−1.34152	4.65803	0.27861
Intercept	−0.04309	0.02539	−0.09286	0.00667	0.08966
Robust MR‐Egger	0.02694	1.72305	−3.35017	3.40404	0.98753
Intercept	−0.02055	0.02501	−0.06957	0.02847	0.41122
Penalized robust MR‐Egger	0.02694	1.72305	−3.35017	3.40404	0.98753
Intercept	−0.02055	0.02501	−0.06957	0.02847	0.41122

* *p* < 0.05.

** *p* < 0.01.

*** *p* < 0.001.

**TABLE 4:** *F* statistics for the selected 17 variants.

ID	*β*	SE	*F* statistic
rs2297792	0.102472	0.0254255	16.24319^a^
rs591120	0.0818076	0.0178911	20.90803^a^
rs2230590	0.0820724	0.0235234	12.17290^a^
rs1052618	−0.0504323	0.0240737	4.38865
rs9438	0.03299	0.0163816	4.05557
rs11755393	0.0430231	0.0163265	6.94411^a^
rs10829163	−0.0385399	0.0164266	5.50460^a^
rs284860	−0.0438767	0.0167741	6.84210^a^
rs11042023	0.0385567	0.0166024	5.39334^a^
rs11555762	0.0452776	0.0185979	5.92705^a^
rs1064608	0.0700331	0.0179628	15.20052^a^
rs12828016	−0.0399526	0.0181992	4.81931
rs1131877	0.0529513	0.0166897	10.06598^a^
rs11071896	−0.045091	0.0216912	4.32128
rs4077410	0.101141	0.0163949	38.05720^a^
rs2306590	−0.0694435	0.0177071	15.38042^a^
rs9891146	−0.0954524	0.0183206	27.14527^a^

^a^ *F* statistic > 5.

**TABLE 5:** Mendelian randomization using selected 13 variants with *F* statistic > 5.

Method	Estimate	SE	95% CI		*p*
Simple median	−1.04025	0.5689752	−2.155417	0.0749252	0.0675076
Weighted median	−1.05527	0.5417685	−2.117118	0.0065751	0.0514356
Penalized weighted median	−1.12237	0.5426655	−2.185979	−0.058769	0.0386154*
IVW	−0.72152	0.4209497	−1.546567	0.1035256	0.0865231
Penalized IVW	−1.02262	0.3830584	−1.773403	−0.271842	0.0075936**
Robust IVW	−0.99143	0.6161391	−2.19904	0.2161807	0.1075944
Penalized robust IVW	−1.05406	0.4051268	−1.848094	−0.260026	0.0092735**
MR‐Egger	1.55658	2.0026993	−2.368638	5.4817989	0.4370165
Intercept	−0.04062	0.0349308	−0.109079	0.0278476	0.2449342
Penalized MR‐Egger	1.55658	2.0026993	−2.368638	5.4817989	0.4370165
Intercept	−0.04062	0.0349308	−0.109079	0.0278476	0.2449342
Robust MR‐Egger	0.44843	1.9773756	−3.427159	4.3240112	0.820596
Intercept	−0.02428	0.0333527	−0.089648	0.0410924	0.4666705
Penalized robust MR‐Egger	0.44843	1.9773756	−3.427159	4.3240112	0.820596
Intercept	−0.02428	0.0333527	−0.089648	0.0410924	0.4666705

* *p* < 0.05.

** *p* < 0.01.

*** *p* < 0.001.

### 3.4 Sensitivity Analyses

To mitigate potential bias from weak instruments, we conducted a secondary MR analysis using only the 13 variants with *F* > 5 (Table 5). Although the estimated effects remained negative, the statistical power was reduced due to fewer instruments. The IVW estimate with the 13‐variant set was nonsignificantly negative (*β* = −0.722, SE = 0.421). Similarly, the simple median (*β* = −1.040, SE = 0.569, 95% CI: −2.155 to +0.075, *p* = 0.068) and weighted median (*β* = −1.055, SE = 0.542, 95% CI: −2.117 to +0.007, *p* = 0.051) methods yielded point estimates consistent with the inverse association. By contrast, several of the more robust or outlier‐resistant methods continued to show significant effects, even with only 13 instruments. The penalized IVW analysis, for instance, strengthened the evidence of a negative effect (β = −1.023, SE = 0.383, 95% CI: −1.773 to −0.272, *p* = 0.0076), indicating that removing the influence of outlier SNPs similarly produced a significant result. Similarly, the penalized robust IVW gave a significant result (*β* = −1.054, SE = 0.405, 95% CI: −1.848 to −0.260, *p* = 0.0093). The penalized weighted median also reached significance (*β* = −1.122, SE = 0.543, 95% CI: −2.186 to −0.059, *p* = 0.0386), reinforcing that the median‐based estimate remains reliably below zero after accounting for outliers. The Egger intercept in this restricted analysis indicated no significant pleiotropic bias (intercept = −0.041, SE = 0.035, *p* = 0.245).

In summary, the majority of MR estimators indicated an inverse relationship between genetically predicted BMI and depression/anxiety symptoms across both sets of analyses. The association was more firmly supported in the analysis using 17 BMI‐associated SNPs, whereas the 13‐SNP analysis revealed a similar trend but with somewhat wider CIs.

## 4 Discussion

Our findings demonstrated that BMI was inversely associated with depression and anxiety symptoms, thereby challenging the conventional expectation that obesity worsens psychological well‐being. Underscoring the complexity of the mind–body relationship, this finding suggests contextual differences in the impact of BMI on mental health.

Although this inverse association between BMI and depression/anxiety may appear counterintuitive, several biological mechanisms may underlie this relationship. Higher BMI in later life may provide greater metabolic reserves and protection against frailty, which have been associated with better mental health outcomes in older adults [24]. Additionally, some evidence suggests altered stress reactivity via the hypothalamic–pituitary–adrenal axis in individuals with higher adiposity, which may influence mood regulation [25]. By contrast, low BMI may reflect malnutrition or underlying illness, which have been linked to poorer mental health outcomes [26, 27]. However, a higher BMI has been robustly linked to elevated levels of inflammatory markers, such as C‐reactive protein and interleukin‐6, which may contribute to the development of depressive and anxiety symptoms through neuroimmune mechanisms [28, 29]. This complex association likely reflects multiple interacting factors, and recent research increasingly emphasizes that the relationship between adiposity and mental health is highly context specific.

Our results are consistent with several studies in older and/or East Asian cohorts reporting similar findings. A cohort study of older adults (aged > 65 years) reported that baseline BMI was negatively correlated with depressive symptom levels, and higher BMI predicted lower depression scores over time during follow‐up [30]. In other words, older people with higher BMI tended to start off less depressed and also experienced a slower increase (or a greater decrease) in depression symptoms, suggesting a protective effect of higher BMI on depressive mood. An epidemiological study observed that obesity was correlated with *lower* anxiety prevalence in older adults but with higher anxiety in younger adults [31]. Furthermore, being underweight (BMI < 18.5) was significantly associated with *increased* risk of anxiety symptoms among Chinese older adults, whereas overweight or obese status did not increase anxiety risk [32]. One study using both observational analyses and MR reported that higher BMI was associated with a lower prevalence of depressive symptoms in a Chinese adult cohort [33]. The study concluded that individuals with obesity had approximately 26% lower odds of depressive symptoms than individuals with normal weight. The MR analysis supported this direction of effect, indicating that genetically elevated BMI *causally* reduced the risk of depression in the East Asian sample. Notably, this protective effect was only observed among East Asian individuals living in Asia but not among those living in Western countries. This finding suggests that cultural or environmental factors can modulate the BMI–depression relationship. These studies demonstrate that an inverse relationship between BMI and mental disorders is plausible, particularly in late adulthood or non‐Western populations. Our research adds to this literature by highlighting a similar inverse association in our study population.

Unlike our findings, many studies have reported that higher BMI is linked to a *greater* incidence of depression and anxiety, not less. A systematic review of longitudinal studies with over 58,000 participants reported that individuals with obesity had 55% higher odds of becoming depressed at follow‐up than those with normal weight [1]. Baseline depression was reported to increase the likelihood of later obesity, supporting a reciprocal association between the two conditions. A large MR study in the UK Biobank reported a significant causal effect of higher BMI on depression risk [13]. Another study also concluded that body fat mass has a direct causal effect on increasing depression risk [6]. Similarly, one study demonstrated a bidirectional relationship between physical activity and depression, further supporting the complex links between lifestyle, adiposity, and mental health [34]. This discrepancy between the literature and our findings warrants further investigation.

Several factors could explain the difference between our results and the positive BMI–mental health association reported in other studies. First, the relationship between BMI and mental health may change with age. In older adults, weight loss and low BMI often accompany frailty, chronic illness, or malnutrition, all of which can contribute to or coincide with depression [35]. By contrast, younger individuals tend to show weight gain during depression (through emotional overeating or medication effects). Therefore, studies focused on youth and mid‐life adults often find obesity to be a risk factor for depression, whereas studies in seniors sometimes find that underweight status is more concerning for mental health [31, 32].

Our study used data from the Taiwan Biobank, which recruits individuals aged 30–70 years across Taiwan. As such, the dataset represents a population of adults and older adults, without the inclusion of adolescents or younger individuals. Moreover, the age distribution in our cohort supports the hypothesis that older adults may drive the inverse association. Among control participants, 30.1% were aged 50–59 and 23.5% were aged ≥ 60 years, indicating that over half of the control group were in the older age brackets. Similarly, in the case group, 32.6% were aged 50–59 and 22.2% were aged ≥ 60 years. These patterns suggest that a substantial portion of the study population consists of middle‐aged and older adults, and the observed inverse correlation could reflect this “jolly fat” effect specific to later life stages.

Sociocultural contexts, especially concerning attitudes toward body weight, can modulate how BMI affects mental well‐being. In environments where a fuller body is culturally acceptable or even desirable, being overweight may carry less stigma and stress, reducing its negative impact on mental health. It might even be associated with a higher socioeconomic status or better access to food, which historically connotes health and security. However, in Western or urbanized settings that idolize thinness, obesity can lead to weight‐related discrimination, body image dissatisfaction, and low self‐esteem, thereby fuelling depression and anxiety. The East Asian MR study illustrates this contrast: higher BMI was linked to lower depression risk among those living in a native Asian context, but this advantage disappeared for East Asians living in Western countries [33]. In the setting of our research, being thin could be associated with poverty or ill health, whereas moderate adiposity could indicate prosperity or robustness, thereby correlating with lower psychological stress. Such sociocultural dynamics would help explain why our results diverge from those in Western populations.

The direction of causality is a critical consideration. Poor mental health may also affect body weight and not necessarily the other way round. Depression often causes loss of appetite, weight loss, and reduced self‐care [36]. Individuals with depression or severe anxiety might lose weight (intentionally or unintentionally), becoming underweight or dropping to a lower BMI category. Therefore, a cross‐sectional study might reveal many individuals with depression or anxiety in the lower BMI group and fewer in the higher BMI group, producing the illusion of an *inverse* association [37], which may mask the long‐term effects of obesity on depression risk. In our study, the mean BMI was comparable between controls and cases (24.2 vs. 24.2, *p* = 0.327; Table 1). However, the MR analysis revealed an inverse association between BMI and depression/anxiety symptoms. Many studies that have identified positive correlations are longitudinal, assessing obesity at baseline and new‐onset depression later [1, 38, 39], thus avoiding this particular bias. Our MR design strengthens the causal inference made in these studies by avoiding reverse causation and largely eliminating confounding factors, because genetic variants are assigned at birth.

Differences in how mental health is defined and measured can also alter the observed association with BMI. Our dataset included self‐reported and screening‐based tools, which may have captured a spectrum of symptom severity. One previous study observed that when it used a broad definition of depression (including self‐reported symptoms), the BMI–depression association was weaker, but when it focused on clinically diagnosed depression, a clearer relationship emerged [33]. Additionally, anxiety and depression were combined as outcomes in our analysis. Because the relationship with BMI differs slightly between these conditions (e.g., some evidence suggests a U‐shaped BMI–anxiety curve [31], but a J‐shaped or linear curve for depression [1]), by grouping them, we obtained an effect that might obscure condition‐specific patterns. Thus, the impact of body weight on mental health can be modified by factors such as age, health status, and culture; this can shift the balance such that *either* end of the BMI spectrum (very low or very high) may carry mental health risks [40].

Our study has several strengths. First, this was a large‐scale investigation with over 100,000 participants, which increased the statistical power and representativeness of our findings. Second, our study incorporated an MR analysis using genetic instruments for BMI (a set of BMI‐associated gene variants). Few other studies have explored the BMI–mental health association using MR in an Asian population. Finally, we addressed an underexplored question in an often‐neglected population, thereby offering insights that have direct public health relevance for that population.

This study has some limitations. First, self‐reported measures and screening tools are subject to bias. Participants may underreport their symptoms, which could lead to misclassification. Second, because our case definition combined depression and anxiety, we could not distinguish the effects on each condition. Future studies should examine these outcomes separately. Finally, we acknowledge the potential for weak instrument bias when selecting an instrument using the lower threshold of *F* > 5.

In conclusion, our findings revealed an inverse relationship between BMI and depression/anxiety symptoms in our cohort of older Taiwanese adults. Our results highlight the complexity of the role of body weight in psychological well‐being and should not be interpreted as advocating for increased body weight as a mental health strategy. Maintaining mental well‐being and physical health, including a healthy weight, is a balancing act, and this balance will likely yield the best outcomes for overall health. Future research and multidisciplinary efforts will help unravel the mechanisms at play and guide evidence‐based recommendations that consider both mind and body in unison.

## Conflicts of Interest

The authors declare no conflicts of interest.

## Data Availability

The data that support the findings of this study are available on request from the corresponding author. The data are not publicly available due to privacy or ethical restrictions.
